# *Plant Communications*: An Open Access Venue for Communicating Diverse Plant Science Discoveries

**DOI:** 10.1016/j.xplc.2019.100018

**Published:** 2019-12-30

**Authors:** Xiaofeng Cui, Bin Han

**Affiliations:** 1 Executive Editor-in-Chief, *Plant Communications*; 2 Editor-in-Chief, *Plant Communications*

With the coming of the new year 2020, we are pleased to invite you to join us in celebrating the birth of a new plant science journal, *Plant Communications*, by reading this editorial. Together with Cell Press, a well-known life science publisher, we are launching *Plant Communications* as an open access journal sister to *Molecular Plant*, one of the leading plant science journals, which has published cutting-edge research for 12 years along with continuously improved services to the global plant science community.

On Earth, no matter where you are living or what you are doing, plants are always central to our well-being, as the Earth is also home to more than 300 000 known species of plants, which have various functions, not only as food but also as integral parts of our living environments and important sources of many industrial products and medicines. Plant sciences, therefore, are critical for us to better understand our lives on earth and to increase our knowledge about the natural sciences. Moreover, plant sciences are important in helping us to find solutions to the grand challenges we are facing, such as food scarcity, global environmental change, and energy crisis. To fulfill our common mission in making the globe better for future generations, we think that all of us should work together to promote plant sciences and to improve scientific publishing and communications. As part of our mission, a prominent goal of *Plant Communications* is to communicate important research advances across all areas of plant sciences as widely as possible and to establish an international platform for academic exchanges, collaborations, and innovations.

*Molecular Plant* has achieved initial success in scientific publishing since its establishment in 2008, with a current impact factor 10.812 that is ranked fourth among 228 JCR-indexed journals with plant science titles. We are happy that *Molecular Plant* has published a lot of landmark discoveries in the past 12 years and is receiving an increasing number of high-quality submissions. However, *Molecular Plant* has been highly selective, and it is regretful that a significant amount of manuscripts reporting interesting findings submitted to *Molecular Plant* had been declined for publication because of the journal's focuses. To better serve the ever-expanding plant science community, we aim to make *Plant Communications* a broad-scope, high-volume plant science journal complementary to *Molecular Plant*, dedicated to publishing exciting research from all disciplines of plant sciences, either fundamental or applied, and to providing professional services to researchers at all career stages. Certainly, *Plant Communications* will inherit all the good characteristics that have evolved in *Molecular Plant*, including straightforward initial submission (uploading a single PDF file with combined text, figures, and supplemental information is recommended), fast and fair reviews with constructive comments, timely and friendly communications with authors and reviewers, rapid online publishing prior to copyediting and typesetting, in-depth scientific editing by professional editors, high-quality production, strong promotion, etc. Nevertheless, *Plant Communications* bears a distinctive feature from *Molecular Plant*: it will be published in a full open-access model, meaning that all published papers will be freely accessible to all for downloading and reading. This not only enables us to rapidly disseminate diverse discoveries from plants to all viewers, but also facilitates the unlimited reuse of the reported sciences for practical applications, something likely to make our daily life better. In addition, both *Plant Communications* and *Molecular Plant* have been using the same Editorial Manager tracking system to process manuscripts. This facilitates the direct transfer of rejected manuscripts from *Molecular Plant* to *Plant Communications*, either pre-review or post-review. To save authors' time in reformatting, *Plant Communications* is also happy to consider the submissions of papers previously reviewed at other high-profile journals without tedious and time-consuming reformatting. When previously reviewed papers report time-sensitive groundbreaking discoveries, *Plant Communications* would like to consider offering authors fast-track evaluations and rapid publication. As the scientific publishing landscape is rapidly evolving, the journal will positively face changes and embrace innovations in submission, peer review, and scholarly publishing in the coming years. For example, *Plant Communications* supports the deposition of large-scale datasets via the FAIR (findable, accessible, interoperable, and reusable) principle to help maximize data reuse and considers the submission or direct transfer of papers previously posted on preprint servers such as bioRxiv (please see https://www.biorxiv.org/about-biorxiv for B2J). We are also keen to get your feedback and suggestions about how we can keep pace with new developments and better serve the community in the coming years. Please do not hesitate to let us know any of your thoughts, whenever.

The academic quality of published papers is crucial for a journal to achieve its goal. At *Plant Communications*, we particularly welcome the submission of review articles containing synthesized analyses/views and forward-looking perspectives, research articles reporting important advances, and resource articles reporting significantly improved technique/methods or community-wide useful resources. In this inaugural issue, we present nine expert reviews, four research articles, and one resource article. Since the release of the whole genomes of *Arabidopsis thaliana*, a dicot model plant, and rice (*Oryza sativa*), one of the staple crops and a monocot model plant, in 2000 and 2002, respectively, plant researchers have widely employed modern molecular and genetic approaches for functional studies of the genes and pathways/networks controlling plant growth, development, and interactions with other organisms and environmental cues. The hidden nature of many genes, non-coding sequences, and interesting biological phenomena in *Arabidopsis* and rice has been uncovered over the past two decades. In a review article, Yuehui He and colleagues discuss genetic and epigenetic regulation of seasonal timing of flowering in *Arabidopsis*, a research topic with tremendous progress since the start of the new century (He et al., 2020). Xin Li and colleagues provide a brief history about plant pathology and review major advances in the understanding of the action modes, downstream signaling pathways, and regulation of plant nucleotide-binding leucine-rich repeat receptors (NLRs), a large family of proteins that recognize pathogen-encoded effectors to trigger immune responses. They also discuss the strategies of engineering of NLR genes for enhancing disease resistance in crops (van Wersch et al., 2020). In another review, Bingkun Lei and Fred Berger summarize research progress on the characterization of histone H2A variants in plants and discuss their roles in the regulation of genome and chromatin functions in *Arabidopsis*, with a particular focus on H2A.Z, which is closely associated with transcriptional regulation (Lei and Berger, 2020). Two research articles in this issue report the precise regulation of AGO1 function in controlling miRNA165/166 activity for maintaining shoot apical meristem and the characterization of three CNGC family members as Ca^2+^-permeable channels required for root hair growth in *Arabidopsis*, respectively (Du et al., 2020, Tan et al., 2020). While active research with model species continues apace, studies focusing on other plant species have gained prominence in the past decade. In this issue, using integrated genetic, molecular, and biochemical approaches, Silvia Gonzali and colleagues discovered that an MYB transcription factor gene, *SlAN2like*, introgressed from wild tomato, positively regulates anthocyanin biosynthesis in purple tomato, and its alternative splicing mutations cause loss of *SlAN2like* function in promoting anthocyanin accumulation in cultivated tomato (Colanero et al., 2020). Yong Wang and colleagues solved the crystal structures of a diterpene glycosyltransferase from *Stevia rebaudiana*, SrUGT76G1, in complex with multiple ligands. SrUGT76G1 catalyzes the formation of economically important natural sweeteners, steviol glycosides. By in-depth structure–function analyses they elucidate the catalytic mechanism of this enzyme and provide general insights into the molecular basis of the substrate specificity of plant diterpene glycosyltransferases (Liu et al., 2020b).

With the advent of cost-effective high-throughput omics technologies, including next-generation RNA and DNA sequencing technologies, the genomes of over 400 plant species have been sequenced so far, and a large number of omics datasets have become available, moving plant research into a new post-genomic era. In a review article, Sheng Luan and colleagues summarize recent progress on the characterization of plant transport proteins in the post-genomic era, discuss the mechanisms and regulation of plant membrane transport, and provide their perspectives on remaining unsolved questions in the field (Tang et al., 2020). Yunbi Xu and colleagues review the bottlenecks and constraints of genomic selection in plants and discuss the strategies of using genomic selection for accelerating breeding processes and enhancing genetic gain (Xu et al., 2020). In another review, Jianbing Yan and colleagues provide an analysis of the processes and genes/loci associated with maize domestication and catalog the available resources for maize research. By surveying the cloned genes controlling important agronomic traits and reviewing their action mechanisms, they propose future directions for maize improvement via genomics and functional genomic routes (Liu et al., 2020a). Plants are associated with diverse species of a huge number of microorganisms, collectively called microbiome or microbiota, which are crucial for the growth and survival of host plants under natural environmental conditions. In a resource article, Yang Bai and colleagues report a host-associated quantitative abundance profiling method for precise assessment of total microbial load and colonization of individual microbiome members relative to host plants. Using this newly developed method they found that increased microbial load and altered species compositions occur in the root microbiome of rice plants exposed to drought stress and of wheat plants with root rot disease (Guo et al., 2020).

In recent years, CRISPR/Cas9-mediated genome editing and synthetic biology technologies have been driving revolutions in biological and biomedical research. Integrating these technologies into plant research is paving the way for creating new crop varieties with enhanced yield and/or better nutritional quality and enabling the synthesis of health-promoting metabolites or even medicines in plants. In a review, Yi Sang and Sanwen Huang focus on plant cytochrome P450s, a superfamily of enzymes involved in a number of catalytic processes, and review previous major strategies and past achievements in P450 engineering and discuss the emerging tools for metabolic engineering based on P450 modifications (Shang and Huang, 2020). Yao-Guang Liu and colleagues discuss the strategies for selecting metabolism-related genes as well as approaches for constructing multigene stacking vectors and genetic transformation into host plants for synthetic metabolic engineering. By reviewing several successful cases, they showcase how plant synthetic metabolic engineering can be used to enhance crop nutritional quality (Zhu et al., 2020). Rik Huisman and Rene Geurts provide a comprehensive review discussing the evolution of nitrogen-fixing nodulation as well as the core genes and signaling pathways essential for nodulation obtained by comparative phylogenomic analyses. They further discuss the strategies toward engineering nitrogen-fixing symbiosis in non-nodulating plant species from evolutionary perspectives (Huisman and Geurts, 2020).

In short, these high-quality review, resource, and research articles published in the inaugural issue of *Plant Communications* cover a wide range of research topics and illustrate the diversity and complexities of plant sciences. We hope you enjoy reading these articles and believe that they nicely showcase the goals and scope of *Plant Communications*: to be a high-quality open access journal with a broad scope for communicating diverse plant science discoveries that will contribute to the development of sustainable agriculture and diverse ecosystems worldwide.

Finally, taking this opportunity, we would like to sincerely thank all of the authors who have contributed papers to this inaugural issue. We also thank all of our advisory and editorial board members and the reviewers, as well as members of our editorial, production, and marketing teams, for their great support from different aspects at various stages: our joint efforts have now culminated in the successful launch of this highly promising new journal! This is an exciting time, at the beginning of a new decade; we look forward to working together with you to support the growth of *Plant Communications* and thereby contribute to the flourishing of global plant sciences in the years ahead.
